# Real-world data of cadonilimab in recurrent or metastatic cervical cancer in China: a multicentric study

**DOI:** 10.3389/fimmu.2025.1611696

**Published:** 2025-07-14

**Authors:** Jian Chen, Haijuan Yu, Yingtao Lin, Dan Hu, Li Liu, Renliang Fan, Jianping Zou, Lele Zang, Yao Lin, Rong Lin, Dezhao Chen, Xiaoying Weng, Fenfang Shen, Shaoyu Wang, Wei Zeng, Qihua Tian, Yun Yi, Yuanfeng Chen, Jinjin Miao, Bo Zhang, Yinxia Zou, Fengming Gao, Rong Lian, Lin Yang, Yang Sun

**Affiliations:** ^1^ Department of Gynecology, Clinical Oncology School of Fujian Medical University, Fujian Cancer Hospital, Fuzhou, China; ^2^ Department of Clinical Medical Research Center, Clinical Oncology School of Fujian Medical University, Fujian Cancer Hospital, Fuzhou, China; ^3^ Department of Pathology, Clinical Oncology School of Fujian Medical University, Fujian Cancer Hospital, Fuzhou, China; ^4^ Department of Oncology, Fujian Medical University Affiliated Nanping First Hospital, Nanping, China; ^5^ Department of Obstetrics and Gynecology, The First Hospital Affiliated to Fujian Medical University, Fuzhou, China; ^6^ Department of Obstetrics and Gynecology, Fujian Provincial Hospital, Fuzhou, China; ^7^ Department of Obstetrics and Gynecology, Fujian Medical University Union Hospital, Fuzhou, China; ^8^ Department of Obstetrics and Gynecology, Gutian Hospital, Ningde, China; ^9^ Department of Gynecology, People’s Hospital Affiliated to Fujian University of Traditional Chinese Medicine, Fuzhou, China; ^10^ Department of Gynecology, Jiangxi Cancer Hospital, Nanchang, China; ^11^ Department of Gynecology, Shunde Women and Children’s Hospital (Maternity and Child Healthcare Hospital of Shunde Foshan), Guangdong Medical University, Foshan, China; ^12^ Department of Obstetrics and Gynecology, Lianyungang Donghai County People’s Hospital, Lianyungang, China; ^13^ Department of Gynecology, Changsha Maternal and Child Health Hospital, Changsha, China; ^14^ Department of Gynecology, Pingxiang Maternal and Child Health Hospital, Pingxiang, China; ^15^ Department of Obstetrics and Gynecology, Huinan County People’s Hospital, Huinan, China; ^16^ Beijing GenePlus Technology Co. Ltd., Beijing, China

**Keywords:** recurrent or metastatic cervical cancer, PD-1, CTLA-4, bi-specific antibody, cadonilimab, real world

## Abstract

**Background:**

Immunotherapy has become a powerful clinical strategy for treating recurrent or metastatic cervical cancer (R/M CC). Cadonilimab, a novel anti-PD-1/CTLA-4 bispecific antibody, has shown substantial clinical benefits in cancer treatment. However, there is no real-world evidence of cadonilimab with a considerable sample size in R/M CC. Hence, we aim to assess the efficacy and safety of cadonilimab in R/M CC patients and explore its potential mechanism.

**Methods:**

This retrospective real-world study examined a sample of R/M CC patients treated with cadonilimab at 13 large academic medical centers in China from July 6, 2022, to October 1, 2023. The outcomes were objective response rate (ORR), disease control rate (DCR), progression-free survival (PFS), overall survival (OS), as well as safety profiles. Additionally, the programmed cell death 1 ligand 1 (PD-L1) was detected by immunohistochemistry to confirm its predictive values. Whole exome sequencing (WES) was also performed to investigate its potential antitumor mechanisms.

**Results:**

Among the 129 patients with measurable disease, the ORR was 38.8%, consisting of complete and partial responses in 8.5% and 30.2% of patients, respectively. The DCR was 72.1%. The median PFS was 12.4 months, while the median OS has not yet been reached. Subgroup analysis showed a numerical trend toward longer median PFS in patients with PD-L1 CPS ≥ 1 compared with CPS < 1 (14.0 vs. 12.8 months; P = 0.235). Moreover, combined therapy of cadonilimab and radiotherapy was identified as an independent prognostic factor for both OS and PFS. The most common grade 3 or worse adverse event was anemia (28 [20.1%]), decreased white blood cell count (24 [17.2%]), and decreased neutrophil count (20 [14.4%]). The most prevalent genetic variant was PIK3CA, highlighting the importance of the PI3K-AKT pathway in the antitumor mechanism of cadonilimab.

**Conclusions:**

Cadonilimab shows an encouraging tumor response rate, with a manageable safety profile in patients with R/M CC. Notably, cadonilimab is also effective for those with PD-L1 CPS <1, suggesting a broad range of application prospects in R/M CC.

**Clinical Trial Registration:**

https://www.clinicaltrials.gov, identifier NCT06140589.

## Introduction

Cervical cancer (CC) is the fourth greatest global burden in terms of both incidence and mortality in women, with the leading cause of cancer death in 37 countries (1). In 2022, Global Cancer Observatory (GLOBOCAN) estimated 661,021 new CC cases and 348,189 CC-related deaths. Notably, China accounts for 22.8% of the worldwide incidence and 16.0% of CC-related mortality, resulting in a tremendous medical burden (2). Although early-stage CC is often amenable to radical surgery or chemoradiotherapy, recurrent or metastatic cervical cancer (R/M CC) patients are incurable and have a dismal prognosis, with a five-year survival rate of only 17% (3). Few effective therapeutic options are left for R/M CC (4). Recent research also emphasizes the urgent need for novel therapeutic strategies in metastatic cancers, as conventional treatments often fail to improve survival outcomes (5).

Recent advances in cancer immunotherapy, which manipulates the immune system to recognize and attack cancer cells (6, 7), have revolutionized the paradigms of CC management (3, 7). Multiple types of immune checkpoint inhibitors (ICIs) targeting programmed death protein-1 (PD-1) and its ligand PD-L1, such as pembrolizumab, camrelizumab, nivolumab, have entered clinical trials successively and exhibited improved efficacy in monotherapy or combination with chemotherapy, radiotherapy, and targeted therapy (8–12). Compared with platinum-based chemotherapy of objective response rate (ORR) ranging from 20% to 30% (13), anti-PD-1 monotherapy could achieve an ORR of 12.2%-33.3%, and reach up to 65.9% when combined with other therapies in late-line treatment of R/M CC (14). Despite this progress, some R/M CC patients still fail to respond to immunotherapy, especially in PD-L1-negative tumors. Hence, considerable strides are urgently needed to improve treatment outcomes.

Dual-targeted immunotherapy is a clinically validated strategy for enhancing antitumor activity compared to anti-PD-1 monotherapy. However, the associated side effects can sometimes be intolerable (15). Cadonilimab (AK-104) is a novel bispecific antibody targeting PD-1 and CTLA-4 with favorable safety profile. It features a unique symmetric tetravalent structure that enhances binding activity within the tumor microenvironment, thereby inhibiting immunosuppressive pathways and augmenting T-cell-mediated responses (16). The emerging class of bispecific antibodies has shown promising results in multiple cancers by enhancing T-cell responses and overcoming tumor immune evasion (17). Cadonilimab became the first bispecific antibody approved for patients with R/M CC who were resistant to platinum-based chemotherapy in China in June 2022, marking a significant advancement in cervical cancer therapy. This approval was based on the COMPASSION-03 trial conducted by Gao et al., in which 111 patients who had failed platinum-based chemotherapy were treated with cadonilimab monotherapy. Cadonilimab achieved an overall response rate (ORR) of 32.3% (32/99), with a median progression-free survival (PFS) of 3.71 months. The ORR in the PD-L1+ cohort was 43.8% (28/64), compared with 16.7% (3/18) in the PD-L1- cohort (18). Additionally, grade ≥3 immune-related adverse events (irAEs) were reported in only 4.5% of patients. Furthermore, modulation of the tumor microenvironment through nanotechnology and molecular engineering may synergize with such bispecific antibodies (19).

Despite such encouraging results, no real-world studies have yet focused on cadonilimab in R/M CC. Against this backdrop, our study presents the first real-world evidence from 13 academic medical centers in China, evaluating the effectiveness and safety of cadonilimab in treating R/M CC. Additionally, we investigate biomarkers such as PD-L1, tumor mutational burden (TMB), and integrated genomic profiling to predict response sensitivity to immunotherapy in R/M CC.

## Materials and methods

### Study design and participants

This multicenter study evaluated the antitumor activity and safety of cadonilimab in R/M CC patients at 13 large academic medical centers in five provinces in China (Fujian, Jiangxi, Guangdong, Hunan, Jiangsu). Patients were included if they had: 1) histologically confirmed R/M CC with pathological types such as squamous cell carcinoma, adenocarcinoma, adenosquamous carcinoma, or neuroendocrine carcinoma; 2) at least one cycle of cadonilimab without restriction on the concurrent use of radiotherapy, chemotherapy, or antiangiogenic therapy. The key exclusion criteria were a history of another malignancy, concurrent malignancies, and incomplete clinical data. All participants were followed up for at least six months after treatment initiation, unless death occurred earlier.

This study was conducted in accordance with the Declaration of Helsinki and Good Clinical Practices Guidelines. The study protocol was approved by the Ethics Committee of Fujian Cancer Hospital (K2023-102-01), with subsequent approvals from all participating centers’ ethics committees. All patients provided written informed consent.

### Treatment

Detailed information on treatment strategies, dosage adjustments, evaluation intervals, and treatment cessation were retrieved from physicians and patients. Based on the physician’s decision, cadonilimab was administered intravenously at 10 mg/kg every three weeks or 6 mg/kg every two weeks. Treatment would be suspended or discontinued due to tumor progression, intolerable AEs, such as severe myocarditis and pancreatitis, or the decision of the patient or physician. In selected cases, patients with ECOG 3 received cadonilimab based on physician judgment and shared decision-making. Their poor performance status was mainly due to reversible tumor-related symptoms, and treatment was initiated with careful monitoring.

### Outcome measures

Responses were assessed by investigators and radiologists according to RECIST version 1.1 (20). The primary outcome measures were ORR and disease control rate (DCR). ORR was defined as the proportion of patients with measurable disease achieving complete response (CR) or partial response (PR). DCR included patients achieving PR, CR, or stable disease (SD). The secondary endpoints were PFS and OS. PFS was measured from initial cadonilimab administration to progressive disease (PD) or death. OS was defined as the time from treatment initiation to death from any cause. Additionally, AEs were recorded in accordance with the National Cancer Institute Common Terminology Criteria for Adverse Events, version 5.0 (21), as were irAEs (22).

### Biomarker exploratory

PD-L1 status was assessed in 74 patients using immunohistochemical staining (detailed in the **Data Supplementary**) and measured by the combined positive score (CPS), defined as the number of PD-L1-positive cells divided by the total number of viable tumor cells, multiplied by 100 (23). CPS ≥1 was considered positive. Whole-exome sequencing was performed on 14 patients, with details provided in the **Data Supplementary**. Sequencing was conducted on the Geneplus-2000 platform (Geneplus, Beijing, China). TMB was classified as high when ≥9 mutations per megabase (mut/Mb) (24). HRD was considered positive with a score of 34 or higher (25).

### Statistical analysis

R version 4.3.2 was used for data analysis. For normal distributions, continuous variables were described as means and ranges. Medians and ranges were used for skewed distributions. Group comparisons were performed using the Chi-square test, Fisher’s exact test, Student’s t-test, or Mann-Whitney U test. The Kaplan-Meier method was used to display the OS and PFS. A P-value <0.05 was considered statistically significant.

## Results

### Patients and treatment

From July 6, 2022, to October 1, 2023, a total of 139 patients with R/M CC were enrolled. Of these, 129 were available for efficacy analysis, while all 139 were included in the safety analysis. The demographic, clinical, and pathological characteristics are summarized in **Table 1**. The median age was 53 years (range: 26-86), with 41.0% at Stage III and 30.2% at Stage IV. The most common metastasis sites were lymph nodes (67, 48.2%), pelvis (46, 33.1%), and lungs (43, 30.9%). Ninety-eight (70.5%) patients were previously treated with platinum-based chemotherapy, and 85 (61.2%) with paclitaxel chemotherapy. Specifically, 36 (25.9%) were previously treated with anti-PD-1 monotherapy, and 42 (30.2%) had received antiangiogenic therapy. As of April 5, 2024, 49 (35.3%) patients still continued with cadonilimab; while 90 (64.8%) patients discontinued due to disease progression (47, 33.8%), treatment-related toxicity (8, 5.8%); financial constraints (27, 19.4%), and patient/physician’s decision (8, 5.8%).

**Table 1:** Baseline characteristics.

Characteristic	Patients (n=139)
Median age, years (range)	53 (26-86)
FIGO stage at initial diagnosis, No. (%)	
IA	2 (1.4)
IB	10 (7.1)
IIA	13 (9.4)
IIB	15 (10.8)
IIIA	1 (0.7)
IIIB	14 (10.1)
IIIC	42 (30.2)
IVA	4 (2.9)
IVB	38 (27.3)
ECOG performance status, No. (%)	
0	15 (10.8)
1	82 (59.0)
2	32 (23.0)
3	10 (7.2)
Histopathological type, No. (%)	
Squamous cell carcinoma	103 (74.1)
Adenocarcinoma	24 (17.3)
Adenosquamous carcinoma	7 (5.0)
Neuroendocrine carcinoma	5 (3.6)
PD-L1 tumor expression status, No. (%)	
Positive (CPS > 1%)	52 (37.4)
Negative (CPS < 1%)	30 (21.6)
Unknown	57 (41.0)
Location of metastases	
Local recurrence only	21 (15.1)
Distant metastasis only	81 (58.3)
Both local recurrence and distant metastasis	30 (21.6)
None*	7 (5.0)
Site of metastases, No. (%)	
Lymph nodes	67 (48.2)
Pelvis	46 (32.6)
Lung	43 (30.9)
Bone	37 (26.6)
Mediastinal	26 (18.7)
Liver	20 (14.4)
Uterus	12 (8.6)
Peritoneum	8 (5.8)
Bladder	8 (5.8)
Skeletal muscle	6 (4.3)
Intestines	6 (4.3)
Pleura	4 (2.9)
Thyroid	3 (2.2)
Others	9 (6.5)
Previous tumor resection	62 (44.6)
Previous Radiotherapy	99 (71.2)
Type of previous systemic cancer therapies, No. (%)	
Previous ICIs	36 (25.9)
Previous AT	42 (30.2)
Previous paclitaxel	85 (61.2)
Previous platinum	98 (70.5)
Previous lines of systemic therapy, No. (%)	
0	32 (23.0)
1	55 (39.6)
2	29 (20.9)
≥3	23 (16.5)
Dose of Cadonilimab, No. (%)	
6mg/kg every 2 weeks	18 (12.9)
10mg/kg every 3 weeks	121 (87.1)
Type of current systemic therapies, No. (%)	
Cadonilimab only	19 (13.7)
Cadonilimab + CT	37 (26.6)
Cadonilimab + RT + CT	20 (14.4)
Cadonilimab + RT + CT + AT	19 (13.7)
Cadonilimab + CT + AT	20 (14.4)
Cadonilimab + AT	18 (12.9)
Cadonilimab + RT + CT + surgery	6 (4.3)
PD-L1 tumor expression status, No. (%)	
Positive (CPS > 1)	52 (37.4)
Negative (CPS < 1)	30 (21.6)
Unknown	57 (41.0)

FIGO, International Federation of Gynecology and Obstetrics; ECOG, Eastern Cooperative Oncology Group performance status; PD-L1, programmed death-ligand 1; CPS, combined positive score; ICI, immune checkpoint inhibitor; AT, antiangiogenic therapy; CT, chemotherapy; RT, radiotherapy. *Six patients received surgery and 1 patient achieved CR before cadonilimab dosing.

Among patients receiving chemotherapy, the most common regimens were paclitaxel combined with carboplatin (n = 76) or cisplatin (n = 16). Less frequently used regimens included albumin-bound paclitaxel plus cisplatin (n = 4), cisplatin plus ifosfamide (n = 1), irinotecan plus carboplatin (n = 1), gemcitabine plus nedaplatin (n = 1), and cisplatin monotherapy (n = 3). Additionally, 57 patients received targeted therapies, including bevacizumab (n = 45) and anlotinib (n = 12). Radiotherapy was administered to the primary tumor and regional lymph nodes in newly diagnosed advanced cases, or to metastatic lesions in patients with recurrent disease. In most cases, radiotherapy was delivered concurrently with cadonilimab, typically within seven days before or after the initiation of immunotherapy. Sequential radiotherapy was administered to 7 patients based on clinical judgment.

Regarding baseline performance status, ECOG scores were 0 in 15 patients (10.8%), 1 in 82 (59.0%), 2 in 32 (23.0%), and 3 in 10 (7.2%). Among the 10 patients with ECOG 3, 9 were evaluable for treatment response. Of these, five showed improvement in performance status during therapy, while 4 remained at ECOG 3 throughout the follow-up period. The objective response rate (ORR) in this subgroup was 33.0%, the disease control rate (DCR) was 55.6%, and the median progression-free survival (PFS) was 5.9 months. As expected, these outcomes were generally poorer than those observed in patients with ECOG 0–2.

### Antitumor activity

**Figure 1:**
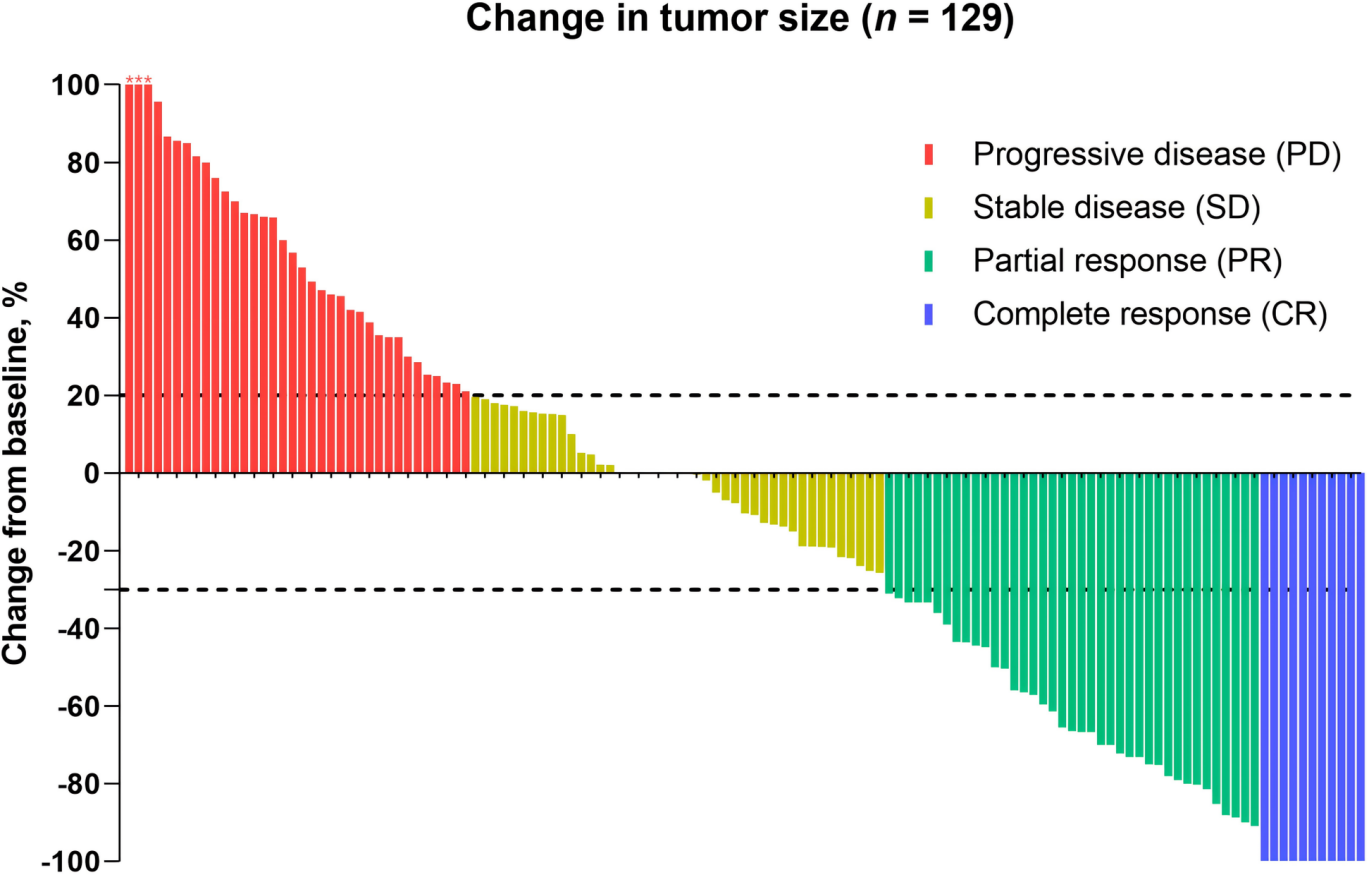
Antitumor activity. Patients who were eligible for the evaluation of treatment efficacy were included (n = 129). The dashed line at +20% change signifies the RECIST version 1.1 cutoff for defining stable disease or progressive disease, whereas the -30% change indicates the cutoff for identifying partial or complete response. *, three patients experienced tumor enlargement exceeding 100%, with increases of 229%, 200%, and 153%, respectively.

**Figure 2:**
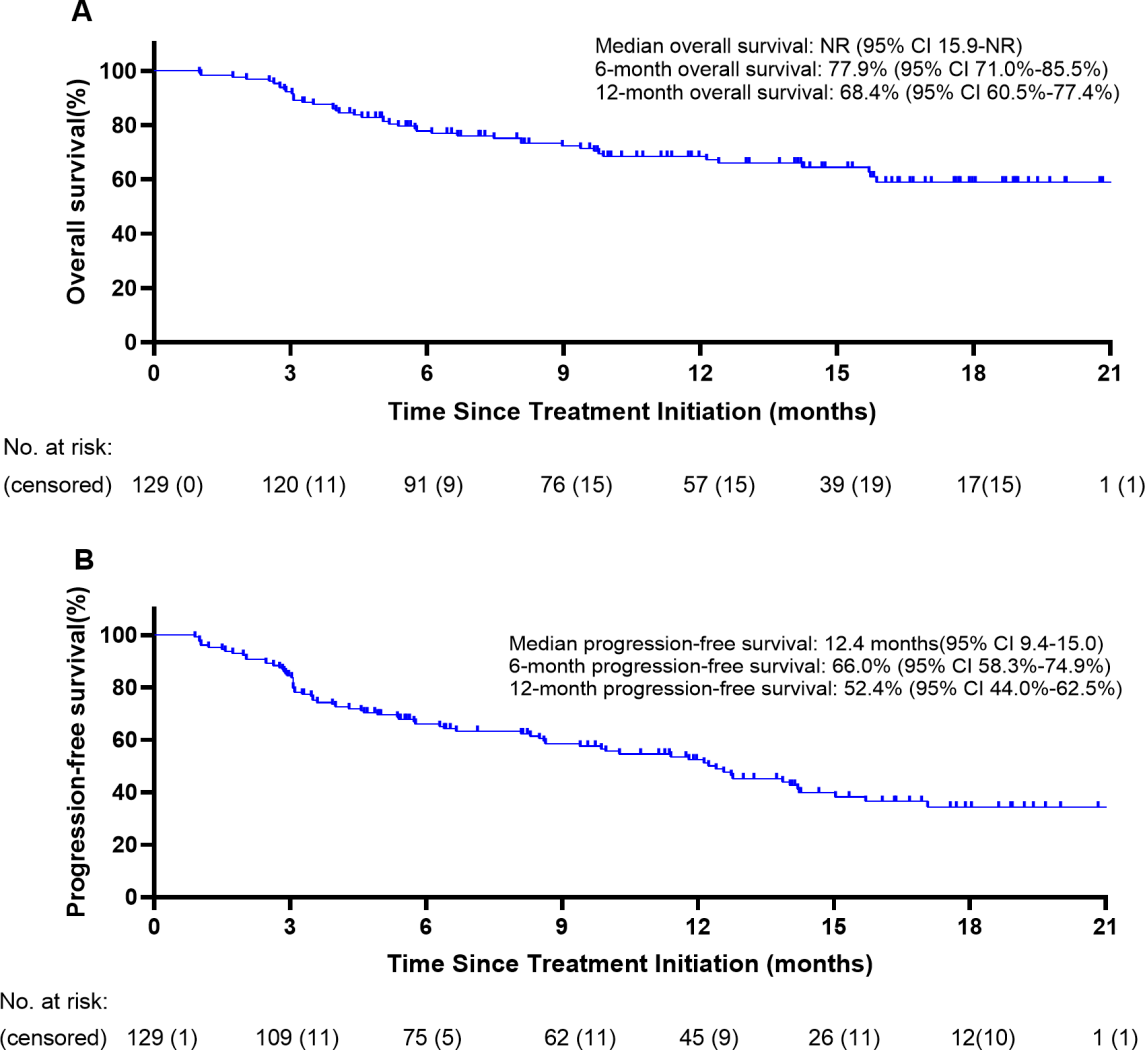
Survival curves. **(A)** Overall survival; **(B)** Progression-free survival.

**Figure 3:**
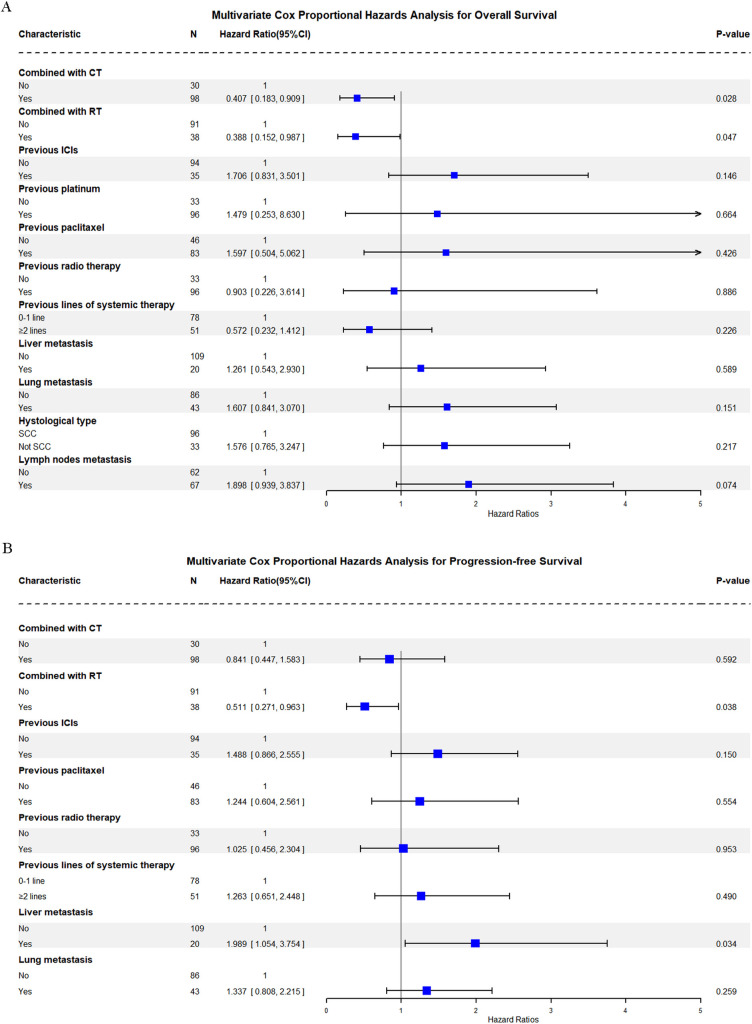
Multivariate Cox proportional hazards analysis for overall and progression-free survival. **(A)** Overall Survival (OS) Multivariate Cox Analysis; **(B)** Progression-free Survival (PFS).

One hundred twenty-nine patients were evaluable for clinical response. Detailed information about therapy modalities and treatment response is presented in **Table 2**. Up to April 5, 2024, the median follow-up time was 10.2 months (range: 1–21 months). There were 11 patients (8.5%) who achieved a best response of CR, 39 (30.2%) with PR, 43 (33.3%) with SD, and 36 (27.9%) with PD as their best overall response (**Figure 1**), resulting in an ORR of 38.8% and a DCR of 72.1% (**Table 3**). At the end of the follow-up period, PD was observed in 72 patients (51.8%), and 45 patients (32.4%) died from the disease. The 6-month and 12-month PFS rates were 66.0% (95% CI, 58.3%-74.9%) and 52.4% (95% CI, 44.0%-62.5%), with a median PFS of 12.4 months (95% CI, 9.4-15.0). The 6-month and 12-month OS rates were 77.9% (95% CI, 71.0%-85.5%) and 68.4% (95% CI, 60.5%-77.4%), with a median OS, which has not been reached (95% CI, 15.9 to not estimable) (**Figure 2**). Furthermore, multivariate analysis identified that combined therapy of cadonilimab and radiotherapy was an independent prognostic factor for both OS and PFS (HR for OS = 0.37 [95% CI, 0.15–0.94], P = 0.036; HR for PFS = 0.51 [95% CI, 0.27–0.96], P = 0.038). Surprisingly, the status of PD-L1 CPS did not significantly impact patient prognosis, indicating the powerful anti-tumor function of cadonilimab in R/M CC patients, regardless of the PD-L1 CPS status (**Figure 3**).

**Table 2:** Best response of different treatments.

Treatment	CR	PR	SD	PD	ORR	DCR
All patients	11 (8.5)	39 (30.2)	43(33.3)	36 (27.9)	50 (38.8^a^)	93 (72.1)
Cadonilimab only	1 (5.3)	2 (10.5)	6 (31.6)	10 (52.6)	3 (15.8)	9 (47.4)
Cadonilimab + CT	3 (8.6)	12 (34.3)	10 (28.6)	10 (28.6)	15 (42.9)	25 (71.4)
Cadonilimab + RT + CT	3 (16.7)	9 (50.0)	4 (22.2)	2 (11.1)	12 (66.7)	16 (88.9)
Cadonilimab + RT + CT + AT	1 (5.6)	6 (33.3)	10 (55.6)	1 (5.6)	7 (38.9)	17 (94.4)
Cadonilimab + CT + AT	2 (10.0)	3 (15.0)	8 (40.0)	7 (35.0)	5 (25.0)	13 (65.0)
Cadonilimab + AT	0 (0)	7 (38.9)	5 (27.8)	6 (33.3)	7 (38.9)	12 (66.7)
Cadonilimab + RT + CT + surgery	1 (100)	0 (0)	0 (0)	0 (0)	1 (100)	1 (100)

CR, complete response; PR, partial response; SD, stable disease; PD, progressive disease; ORR, objective response rate; DCR, disease control rate; CT, chemotherapy; AT, antiangiogenic therapy; RT, radiotherapy; a, Due to rounding, the sum of individual response rates (CR and PR) may not equal the total ORR exactly.

**Table 3:** Antitumor activity and treatment outcomes.

Outcome	Patients (N=129)^a^
ORR. N (%)	50 (38.8^b^)
95% CI	30.2-47.3
DCR. N (%)	93 (72.1)
95% CI	63.6-79.1
Best overall response. N (%)	
CR	11 (8.5)
PR	39 (30.2)
SD	43 (33.3)
PD	36 (27.9)
Treatment ongoing. N (%)	
Yes	42 (32.6)
No	87 (67.4)
Reason for discontinuing treatment. N (%)	
Disease progression	46 (35.7)
Financial constraints	26 (20.2)
Patient decision	8 (6.2)
Toxicity	7 (5.4)
Disease progression. N (%)	
Yes	70 (54.3)
No	59 (45.7)

ORR, objective response rate; CI, confidence interval; DCR, disease control rate; CR, complete response; PR, partial response; SD, stable disease; PD, progressive disease. a, Objective response was assessed in 129 participants who met the RECIST criteria for measurable disease; b, Due to rounding, the sum of individual response rates (CR and PR) may not equal the total ORR exactly.

### Safety

During treatment, 133 (95.7%) patients experienced treatment-related adverse events of any grade, with no unexpected AEs recorded. Fifty-six (40.3%) patients developed grade 3–4 adverse events (**Table 4**). The most common grade 3 or worse adverse events were anemia (28 [20.1%]), decreased white blood cell count (24 [17.2%]), and decreased neutrophil count (20 [14.4%]). IrAEs occurred in 79 (56.8%) patients, with the most frequent being hypothyroidism (31 [22.3%]) and rash (20 [14.4%]), followed by hypercorticism (15 [10.8%]) and hyperthyroidism (14 [10.1%]). Seven (5.3%) patients developed grade 3 severe irAEs during treatment, including myocarditis (3 [2.2%]), pancreatitis (2 [1.4%]), hypocorticism (1 [0.7%]), hepatitis (1 [0.7%]), rash (1 [0.7%]), and myositis (1 [0.7%]). Notably, most grade 3 or higher irAEs were successfully managed with therapeutic interventions.

**Table 4:** Adverse events, N (%).

Adverse Event	Total	Grade 1	Grade 2	Grade 3	Grade 4
Anemia	100 (71.9)	33 (23.7)	39 (28.1)	26 (18.7)	2 (1.4)
Hypoalbuminemia	98 (70.5)	75 (54.0)	23 (16.5)	0 (0)	0 (0)
Decreased white blood cell count	78 (56.1)	26 (18.7)	28 (20.1)	17 (12.2)	7 (5.0)
Decreased neutrophil count	63 (45.3)	21 (15.1)	22 (15.8)	9 (6.5)	11 (7.9)
Hyperglycemia	52 (37.4)	48 (34.5)	1 (0.7)	3 (2.2)	0 (0)
Hypertriglyceridemia	50 (36.0)	34 (24.5)	15 (10.8)	1 (0.7)	0 (0)
Hypercholesterolemia	39 (28.1)	31 (22.3)	8 (5.8)	0 (0)	0 (0)
Proteinuria	50 (36.0)	36 (25.9)	14 (10.1)	0 (0)	0 (0)
Hypokalemia	44 (31.7)	31 (22.3)	6 (4.3)	5 (3.6)	2 (1.4)
Hypomagnesaemia	42 (30.2)	39 (28.1)	1 (0.7)	2 (1.4)	0 (0)
Decreased platelet count	41 (29.5)	19 (13.7)	10 (7.2)	7 (5.0)	5 (3.6)
Hypophosphatemia	33 (23.7)	30 (21.6)	1 (0.7)	2 (1.4)	0 (0)
Hepatic function abnormal	30 (21.6)	20 (14.4)	4 (2.9)	6 (4.3)	0 (0)
Increased aspartate aminotransferase	30 (21.6)	19 (13.7)	5 (3.6)	6 (4.3)	0 (0)
Hyponatremia	27 (19.4)	18 (12.9)	9 (6.5)	0 (0)	0 (0)
Increased alanine aminotransferase	26 (18.7)	21 (15.1)	3 (2.2)	2 (1.4)	0 (0)
Increased troponin I	16 (11.5)	13 (9.4)	0 (0)	3 (2.2)	0 (0)
Increased blood bilirubin	16 (11.5)	12 (8.6)	3 (2.2)	1 (0.7)	0 (0)
Nausea	15 (10.8)	10 (7.2)	5 (3.6)	0 (0)	0 (0)
Hyperthyroidism	14 (10.1)	5 (3.6)	9 (6.5)	0 (0)	0 (0)
Diarrhea	12 (8.6)	7 (5.0)	2 (1.4)	3 (2.2)	0 (0)
Increased conjugated bilirubin	12 (8.6)	7 (5.0)	3 (2.2)	2 (1.4)	0 (0)
Infection	11 (7.9)	0 (0)	0 (0)	8 (5.8)	3 (2.2)
Blood creatinine increased	9 (6.5)	5 (3.6)	3 (2.2)	1 (0.7)	0 (0)
Pyrexia	7 (5.0)	4 (2.9)	3 (2.2)	0 (0)	0 (0)
Increased unconjugated bilirubin	6 (4.3)	5 (3.6)	1 (0.7)	0 (0)	0 (0)
Increased blood creatine phosphokinase	5 (3.6)	2 (1.4)	2 (1.4)	1 (0.7)	0 (0)
Constipation	5 (3.6)	5 (3.6)	0 (0)	0 (0)	0 (0)
Vomiting	5 (3.6)	4 (2.9)	1 (0.7)	0 (0)	0 (0)
Hypernatremia	4 (2.9)	4 (2.9)	0 (0)	0 (0)	0 (0)
Hyperkalemia	2 (1.4)	0 (0)	2 (1.4)	0 (0)	0 (0)
Ileus	1 (0.7)	0 (0)	0 (0)	1 (0.7)	0 (0)
Immune-related adverse events					
Immune-mediated hypothyroidism	31 (22.3)	16 (11.5)	15 (10.8)	0 (0)	0 (0)
Immune-mediated Rash	20 (14.4)	14 (10.1)	5 (3.6)	1 (0.7)	0 (0)
Immune-mediated hypocorticism	15 (10.8)	12 (8.6)	2 (1.4)	1 (0.7)	0 (0)
Immune-mediated myocarditis	6 (4.3)	0 (0)	3 (2.2)	3 (2.2)	0 (0)
Immune-mediated pancreatitis	3 (2.2)	0 (0)	1 (0.7)	2 (1.4)	0 (0)
Immune-mediated hepatitis	1 (0.7)	0 (0)	0 (0)	1 (0.7)	0 (0)
Immune-mediated myositis	1 (0.7)	0 (0)	0 (0)	1 (0.7)	0 (0)
Immune-mediated enteritis	1 (0.7)	0 (0)	1 (0.7)	0 (0)	0 (0)
Immune-mediated pneumonitis	1 (0.7)	0 (0)	1 (0.7)	0 (0)	0 (0)

### Biomarkers and potential mechanism

PD-L1 expression was evaluable in 74 patients, and 46 patients had a PD-L1 CPS ≥1. Individuals with a PD-L1 CPS ≥1 trended to exhibit a higher ORR (26 of 46, 56.5%) compared to those with a CPS <1 (10 of 28, 35.7%). However, this difference was not significant (P = 0.082). Moreover, PFS did not differ significantly between these two groups (P = 0.235). The median PFS was longer in the PD-L1 CPS ≥1 group of 14.0 months (95% CI: 12.1-NR) compared to 12.8 months (95% CI: 6.3-NR) for the PD-L1 CPS <1 group. These results suggest that cadonilimab may also be effective in patients with PD-L1 CPS <1. Further investigations are merited to determine its role in the PD-L1 CPS <1 subgroup.

**Figure 4:**
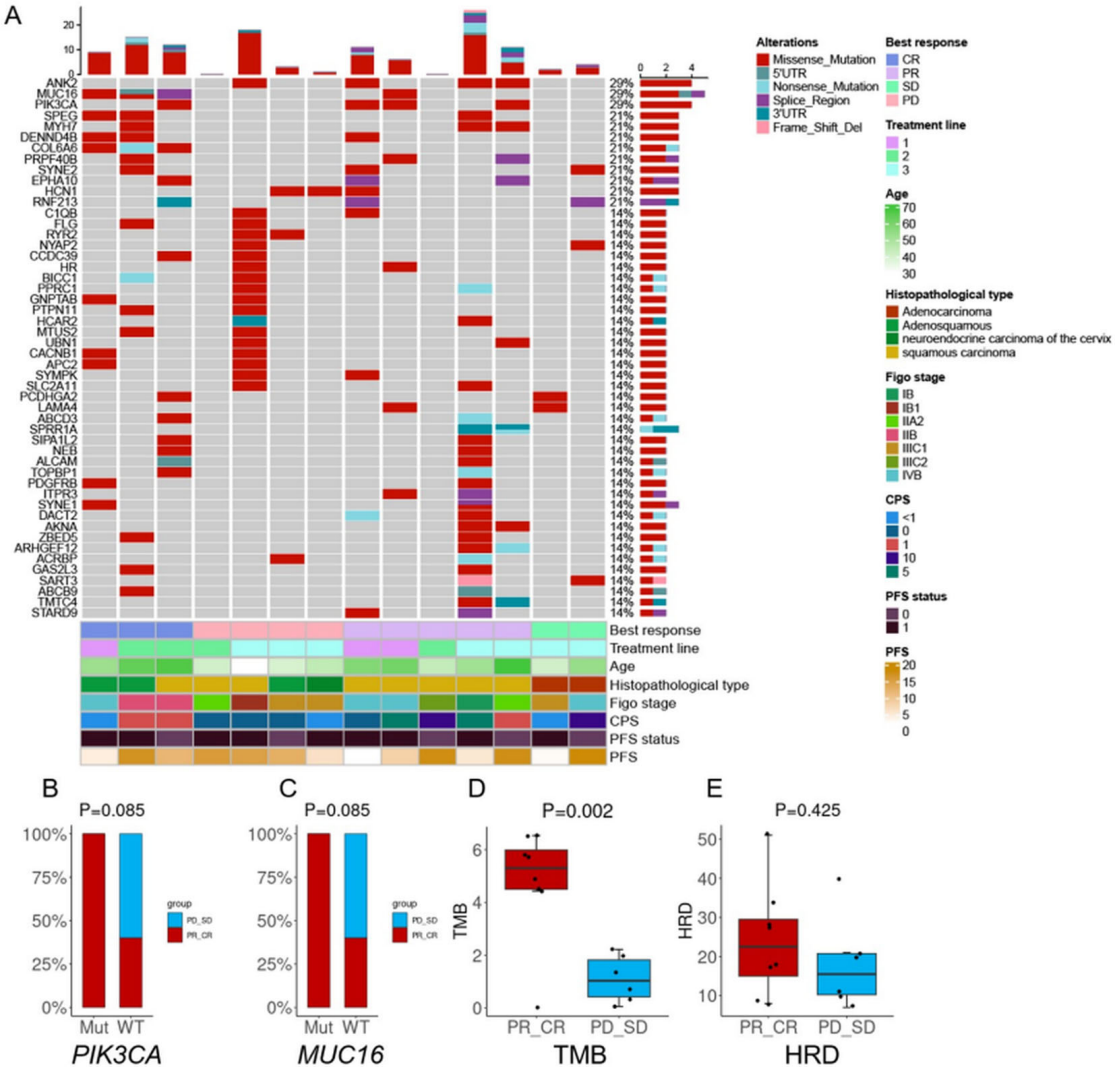
Biomarker analyses. **(A)** OncoPrint of functional driver mutations in 14 patients with cervical cancer. Rows represent genes; columns represent samples. Glyphs and color coding display genomic alterations (mutations, copy number alterations, changes in gene expression) and best response, treatment line, age, histopathological type, FIGO stage, combined positive score (CPS), and progression-free survival (PFS); **(B)** Comparison of tumor mutational burden (TMB) by treatment response; **(C)** Comparison of homologous recombination deficiency (HRD) by treatment response; **(D)** Treatment response in patients with altered vs. wild-type PIK3CA; **(E)** Treatment response in patients with altered vs. wild-type MUC16. Abbreviations: CR, complete response; PR, partial response; SD, stable disease; PD, progressive disease.

Whole-exome sequencing was performed on 14 patients using qualified control samples. The most frequently altered genes were PIK3CA, MUC16, and ANK2, each identified in 4 patients (4 of 14, 28.6%) (**Figure 4A**). All PIK3CA mutations were missense variants located in exon 9, including E542K (c.1624G>A) and E545K (c.1633G>A), and were detected only in patients with complete or partial response. No PIK3CA mutations were observed in patients with stable or progressive disease (**Figures 4B, C**). KEGG pathway enrichment analysis showed that these mutations were enriched in the PI3K-AKT signaling pathway, often co-occurring with COL6A6 alterations. Moreover, significantly higher median TMB values were detected in patients who achieved CR or PR compared to those with SD or PD (4.8 vs. 1.1, P=0.002; **Figure 4D**). ORR exhibited no significant differences between participants with HRD-positive (66.7%; n=3) and HRD-negative ones (54.5%; n=11) (P = 0.615; **Figure 4E**).

## Discussion

Cadonilimab yielded favorable response rates without compromising safety profiles in R/M CC management. To our knowledge, this multicenter study represents the first real-world assessment of cadonilimab in R/M CC patients. Cadonilimab exhibited an ORR of 38.8% and a DCR of 72.1%. Among those with PD-L1 CPS ≥1, the ORR improved to 56.5%, and the median PFS reached 14.0 months. Notably, patients with PD-L1 CPS <1 also demonstrated significant benefits, with an ORR of 35.7% and a median PFS of 12.8 months, indicating cadonilimab could also provide encouraging anti-tumor activity even in PD-L1 CPS <1 subgroup. Moreover, only 56 (40.3%) patients developed grade 3–4 adverse events in our study, comparable to or lower than the previous study (14), indicating its well-tolerated nature. These results are consistent with prior findings that novel immune agents can offer a favorable safety profile compared to traditional checkpoint inhibitors (26). However, the long-term evaluation of cadonilimab needs an expanded sample and further follow-up.

Cadonilimab treatment led to significantly longer PFS (12.4 months) than cisplatin-based combination therapy (5.8 months), followed by non-platinum drugs (2–3 months) among R/M CC patients (27). Compared with the investigator’s choice of cisplatin-based chemotherapy, cadonilimab treatment resulted in a 9.7% higher ORR (27). Similarly to our study, the COMPASSION-03 study of the CC cohort also demonstrated cadonilimab with a high ORR of 32.3% in R/M cases (18). Compared with another generic drug (QL1706), cadonilimab also showed higher ORR in CC treatment (28). Hence, the anti-PD-1/CTLA-4 bispecific antibodies, like cadonilimab, have become an important component of the treatment regimen for R/M CC. Moreover, compared to dual PD-1 and CTLA-4 checkpoint blockade combinations in CC treatment, such as the CheckMate 358 study (29) and a Phase II trial of balstilimab and zalifrelimab combination (NCT03495882) (30), cadonilimab showed a more favorable anti-tumor power with higher ORR and lower toxicity, underscoring its enormous potential in CC management.

The significant antitumor activity of cadonilimab is likely due to its tetravalent design. Preclinical studies have suggested its tetravalent structure could enhance the binding avidity of high densities of PD-1 and CTLA-4, leading to increased immune responses to antitumors (16). Mechanistically, blocking the binding of PD-1 and PD-L1 could maintain the active status of tumor-reactive T cells, which would be becoming inactivated after persistent chronic stimulation. On the other hand, CTLA-4 blockade could activate effector T cells and inactivate regulatory T cells, thereby enhancing antitumor immunity. The unique complementary mechanisms of blockade of the PD-1 and CTLA-4 pathways underlie the improved antitumor activities of cadonilimab (31).

An increasing body of evidence also supported the synergistic effect of radiotherapy and immunotherapy in treating malignant tumors, such as non-small cell lung cancer (32) and esophageal or gastroesophageal junction cancer (33), as it can eliminate the primary tumors and induce host immunity to control distant metastases (34). Consistent with previous studies, we found that patients treated with cadonilimab in combination with radiotherapy demonstrated a higher ORR of 52.6% and a DCR of 86.8%, further identifying the combination of radiotherapy as an independent prognostic factor for both OS and PFS in R/M CC. Previous studies have also confirmed that combination strategies, including immunotherapy and radiation, can enhance anti-tumor efficacy via synergistic mechanisms (35). Preclinical studies show that radiotherapy can induce tumor cell death and antigen release, enhancing the immune response by altering the tumor microenvironment and making tumor cells more recognizable to immune cells (36). In addition, ICIs could promote tumor vascular normalization in a T-cell-dependent manner through interferon (IFN)-mediated signaling between T cells and endothelial cells, improving tissue perfusion and reducing intratumoral hypoxia and acidosis, thereby sensitizing tumors to ionizing radiation (37). Hence, the combination of cadonilimab and radiotherapy has shown significant potential in treating R/M CC and might emerge as an important therapeutic strategy in the future.

The safety profile of cadonilimab was consistent with that previously reported for the drug and combined therapy in patients with different tumor types (38–40). In our study, cadonilimab, combined with other therapies, generally reflects a manageable safety profile with mostly grade 1–2 TRAEs, even under the circumstance of prolonged exposure to cadonilimab. It is worth noting that the incidence of grade 3–4 irAEs among participants treated with cadonilimab monotherapy was only 31.6% (6/19), comparable to the 28% reported in the COMPASSION-03 study and lower than MEDI5752 (38%), another bispecific anti-PD-1/CTLA-4 antibody (18, 41). When combined with other treatments, only 46.6% of patients experienced grade ≥3 TRAEs, which is also lower than the reported rate of 73.3% (18), underscoring cadonilimab’s favorable safety profiles. Mechanistically, cadonilimab’s favorable safety profile is likely attributed to its unique Fc-null design. The Fc-null design inhibits binding to Fc receptors, significantly reducing antibody-dependent cellular cytotoxicity (ADCC), antibody-dependent cellular phagocytosis (ADCP), and the release of interleukins-6 and -8 (42). Immune-related toxicity profiles might also be influenced by monocyte/macrophage repolarization, a mechanism gaining attention in cancer immunotherapy (43). These properties collectively contribute to the low toxicity observed in clinical settings.

In-depth analysis of the PIK3CA mutation landscape in our cohort revealed that all alterations were restricted to canonical helical domain hotspots—E542K and E545K—which are among the most frequently reported oncogenic mutations in solid tumors, including cervical cancer (44). These mutations are known to promote constitutive activation of the PI3K-AKT pathway by relieving inhibitory interactions with the p85 regulatory subunit, thereby enhancing downstream proliferative and survival signaling (45). Both E542K and E545K have been implicated in increased immune evasion and tumor aggressiveness (46). Furthermore, KEGG pathway enrichment analysis in our cohort demonstrated that *PIK3CA* mutations often co-occurred with *COL6A6* alterations and were significantly enriched in the PI3K–AKT signaling pathway. This pathway not only drives cell cycle progression and metabolic adaptation but also shapes the tumor immune microenvironment by regulating apoptosis resistance, cytokine secretion, and immune cell recruitment (47, 48). Notably, our findings are consistent with a prior report by Xu et al. (49), which demonstrated that patients harboring *PIK3CA* mutations exhibited a significantly higher objective response rate (ORR) to anti-PD-1 therapy (91.7%) compared to those with wild-type *PIK3CA* (46.2%; *P* = 0.012). In our study, all four patients with *PIK3CA* mutations responded to cadonilimab (CR or PR), whereas no mutations were detected in the non-responder group. Although this trend did not reach statistical significance (*P* = 0.084), likely due to the limited sample size, the observed pattern suggests a potential predictive role of *PIK3CA* mutations for immunotherapy responsiveness in selected patients. These findings are exploratory in nature and warrant validation in larger, independent cohorts. Emerging evidence also suggests that traditional compounds, including herbal derivatives, may modulate tumor immunogenicity and complement checkpoint-based approaches (50). Furthermore, future mechanistic studies are needed to explore whether aberrant PI3K–AKT pathway activation influences cadonilimab sensitivity by modulating immune evasion or the tumor microenvironment.

Of note, to our knowledge, this study represents the largest real-world data set to date evaluating cadonilimab as a therapeutic modality in patients with R/M CC. On the one hand, compared to the limited use of ICIs targeting PD-1 in PD-L1-negative populations with a low ORR ranging from 0-16.7% (9, 51–54). Cadonilimab demonstrated significant antitumor activity even in a PD-L1-negative population, with an ORR of 35.7% and an mPFS of 12.8 months, indicating the broad application of cadonilimab in the PD-L1-negative population. On the other hand, 8.5% of the population achieved CR, giving us hope that recovery from disseminated tumors and maintaining a normal life are no longer a luxuries for late-stage CC patients in the era of immunotherapy. Further in-depth research and fundamental experiments are necessary to explore the potential roles of cadonilimab in the low PD-L1 expression subgroup and its antitumor mechanism.

Despite the encouraging results, several limitations should be acknowledged. First, this was a retrospective study conducted in China, which led to selection bias and limited generalizability. Second, the long-term benefits and late toxicities were unavailable due to the relatively short follow-up time. Third, many challenges remain in the clinical use of cadonilimab due to financial constraints. However, with growing real-world evidence, we believe cadonilimab will soon be covered by Chinese medical insurance and benefit more R/M CC patients. Moreover, treatment regimens in our study were heterogeneous, and baseline characteristics varied across subgroups. Due to the limited sample size, we did not perform propensity score matching or multivariable adjustment. Therefore, subgroup analyses—such as those based on PD-L1 CPS—should be considered exploratory and interpreted with caution. Lastly, the limited number of patients who underwent PD-L1 testing and whole-exome sequencing reduced the statistical power to assess the predictive value of biomarkers such as PD-L1 expression, TMB, and PIK3CA mutations. These exploratory findings should be interpreted with caution and validated in larger prospective studies.

## Conclusion

In sum, R/M CC is a life-threatening disease with limited treatment options available. In our study, cadonilimab demonstrated promising efficacy with acceptable safety profile, even in the low PD-L1 population. Cadonilimab has the potential to become a new standard of care in treating CC. Further investigations involving larger-scale randomized clinical trials and real-world studies are warranted.

## Data Availability

The datasets generated and/or analyzed during the current study are not publicly available due to patient privacy and institutional restrictions. De-identified data may be available from the corresponding author on reasonable request and with appropriate institutional approvals.
