# Supplementary material for: Real-world data of cadonilimab in recurrent or metastatic cervical cancer in China: a multicentric study
Source: Front Immunol. 2025 Jul 14;16:1611696. doi: 10.3389/fimmu.2025.1611696 (PMC12301402; doi:10.3389/fimmu.2025.1611696)
Supplement: Supplementary file 1 [file DataSheet1.docx]

**Biomarker exploratory**

**PD-L1 status test**

All samples were fixed in 4% neutral formalin solution and routinely embedded in paraffin. Immunohistochemical staining was conducted using the fully automated BenchMark XT system (Roche, Switzerland) with the EnVision two-step method. The procedure strictly followed the manufacturer's protocols for both the instrument and the antibodies. DAB was used for chromogenic detection, followed by hematoxylin counterstaining, and the slides were examined under a microscope. Positive and negative controls were included. PD-L1 (clone 28-8, dilution 1:2000, Abcam) was defined as negative for CPS < 1 and positive for CPS ≥ 1.

**whole-exome sequencing**

The whole-exome sequencing was undertaken in individuals with their consent to analyze genetic differences. DNA was extracted from formalin-fixed paraffin-embedded (FFPE) tissues and peripheral blood utilizing the QIAamp DNA FFPE Tissue Kit and the DNeasy Blood & Tissue Kit, both of which were products of QIAGEN. The construction of sequencing libraries is facilitated using the KAPA Hyper Prep Kit (KAPA Biosystems), in accordance with a refined protocol provided by the manufacturer. Sequencing libraries were constructed with the KAPA Hyper Prep Kit (KAPA Biosystems) following the manufacturer’s refined protocol. Sequencing was performed on the Geneplus-2000 platform (Geneplus, Beijing, China), with tumor samples achieving an average coverage depth of approximately 300x and control samples an average depth of about 150x.

**Tumor mutational burden and homologous recombination deficiency**

Tumor mutational burden (TMB) and homologous recombination deficiency (HRD) were assessed by whole-exome sequencing using DNA isolated from formalin-fixed paraffin-embedded slides of tumor and whole blood control samples. TMB is quantified as the number of non-synonymous somatic mutations per megabase (mut/Mb) of the coding interval. A TMB of 9 mut/Mb or higher is categorized as high TMB (TMB-H). To assess genomic instability, we utilized the scarHRD software, which analyzes loss of heterozygosity (LOH), telomeric allelic imbalance (TAI), and large-scale state transitions (LST) across the genome. These assessments contribute to the Genomic Instability Score (GIS), which is then used to calculate the overall HRDscore. An HRD score of 34 or higher is considered positive, indicating significant genomic instability.
